# Social Determinants of Food Literacy in Iranian Adult Population: A Cross‐Sectional Study

**DOI:** 10.1002/fsn3.70044

**Published:** 2025-03-07

**Authors:** Mozhgan Moshtagh, Mojtaba Daneshvar, Gholamreza Ghaedamini Harouni

**Affiliations:** ^1^ Birjand University of Medical Sciences Birjand Iran; ^2^ Tehran University of Medical Sciences Tehran Iran; ^3^ University of Social Welfare and Rehabilitation Science Tehran Iran

**Keywords:** cross‐sectional study, food literacy, Iranian adults, social determinants

## Abstract

This research aimed to evaluate the social determinants influencing food literacy among Iranian adults. This cross‐sectional investigation was executed during the period spanning from 2023 to 2024, targeting voluntary adult participants (aged 18–65) residing across four provinces (comprising 547 individuals). The study employed questionnaires to assess three behavioral aspects related to food, participants' attitudes toward social media, and their levels of food literacy. The outcomes derived from the regression analysis indicated that various factors, including female gender, marital status, extensive use of social media, advanced age, elevated educational attainment, and a favorable attitude toward social media, were significantly correlated with enhanced food literacy levels. Collectively, these predictors elucidated 16.2% of the variance observed in food literacy. This study highlights Iran's significant social determinants of food literacy. Promoting informed eating practices across diverse demographic groups may be useful in achieving healthier communities in Iran. Future studies should investigate the underlying mechanisms for designing interventions. Performing these investigations within specific contexts could help determine effective strategies for enhancing nutritional knowledge.

## Graphical abstract

Promoting informed eating practices across diverse demographic groups may be useful to achieve healthier communities in Iran. Understanding sociodemographic factors such as gender, age, marital status, education using social media and attitude toward it is crucial for developing targeted interventions aimed at improving Iranian food literacy. Performing future investigations within specific contexts could help determine effective strategies for enhancing nutritional knowledge.

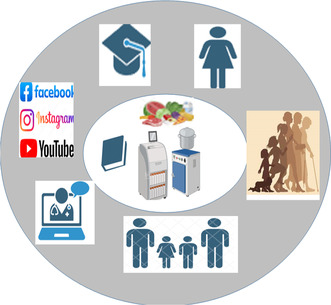

## 1 Introduction

Nutrition is identified an influential factor in public and individual health as dietary habits have significant role in prevention of noncommunicable diseases and managing their drawbacks (Forray et al. 2023).

Healthy eating and food habits are complex behaviors influenced by personal characteristics and preferences, culture, and environment (Mozaffarian 2016). These conditions are changing in accordance with time, place, culture, and living situations. In the current era, cultural transition and preferences is considered a threatening factor of food security especially for low‐income and under developed countries (Fathelrahman et al. 2016).

Food utilization and food stability are two dimensions of food security that are crucial for promoting societal health and welfare (Fathelrahman et al. 2016). Food security could affect the nutrition status and health of individuals based on their level of access to safe and sufficient foods (McKay et al. 2019). Therefore, modified behaviors related to foods is the best policy to improving food security as altering the culture and context is arduous and lengthy or even impossible (Thomas et al. 2019).

Using social media could be used for promoting cultures advocating maintaining natural resources and food sustainability through improving societal knowledge, attitudes, and practices defined as food literacy. In this regard, food literacy concept has been proposed as an action to improving utilization and stability of foods by maintaining natural resources and environments in different contexts (Thomas et al. 2019). Food literacy (FL) refers to a process of decision, selection, readiness, and consumption of proper foods to meet human needs using accurate knowledge, abilities and doing actions on the food substances (Vidgen and Gallegos 2014). The administration of dietary patterns, the formulation of decisions regarding food selection, and the behaviors associated with dietary practices are individual obligations within the familial framework (Gibbs and Chapman‐Novakofski 2012). Enhancing dietary patterns and fostering healthy habits within familial units may contribute to the promotion of appropriate eating behaviors and sustainable dietary practices. Heterogeneous populations may exhibit varying food literacy and perspectives regarding acceptable and secure dietary practices, and their access to precise information, as well as their capacity to perceive and interpret such information, is not uniformly distributed (Azevedo Perry et al. 2017).

Understanding associated factors can aid the change process and lead to positive outcomes. Research indicates that gender, age, income, education, and BMI affect food literacy and attitudes toward a healthy diet. (Banna et al. 2022; Wijayaratne et al. 2018). In the study by Soon et al. (2021), education and gender were not associated with food safety knowledge (Soon et al. 2021). Additionally, a study indicated that media such as television can greatly impact knowledge about food safety (Bolek 2020).

FL concept can motivate individuals to adhere to sustainable diets that could help in achieving food security as well as protecting ecosystems and natural supplies particularly in under‐resourced contexts (Rosas et al. 2021; Whittall et al. 2023). Iran is a vulnerable context in terms of probability of food insecurity and food illiteracy in the coming years owing to climate and ecologic instability (Fathelrahman et al. 2016). Evaluating social determinants of food literacy in Iran may illuminate the properties that could help in finding feasible practices within a different context and culture. The current research may be useful for policymaking and planning actions to achieve sustainable development goals in the food system. Thus, this study decided to assess social determinants of food literacy in Iranian adult population.

## 2 Methods

### 2.1 Study Design

This cross‐sectional study was conducted between 2023 and 2024. Data were collected after receiving approval from the ethics committee of Birjand University and completing the validation process of the food literacy questionnaire (IR.BUMS.Rec.1401.069).

Tehran, the capital city of Iran, serves as a domicile for a heterogeneous array of social strata and migratory populations originating from an assortment of cities and cultures, rendering it an appropriate locale for the examination of the correlation between sociocultural determinants and food literacy. For the purposes of this investigation, we not only incorporated Tehran but also randomly selected the eastern region of the nation, with a particular emphasis on three provinces that were historically unified but have since been delineated into northern, central (Razavi), and southern segments of Khorasan. The selection of these provinces was predicated upon their ethnic heterogeneity, abundant traditional practices, and diverse ecological and climatic conditions. In order to recruit participants from each of the designated provinces, we implemented a probability proportional to size sampling methodology.

### 2.2 Study Population

Residents of the provinces Tehran, Razavi Khorasan, and North and South Khorasan who tended to participate in the study and gave written informed consent were interviewed using study tools. The criteria included age above 18 years, being literate and able to understand and answer the questions. A total of 700 individuals were invited to the survey, of which 547 accepted and were interviewed.

### 2.3 Data Collection

The necessary information was obtained using questionnaires. Three questions were asked about food‐related responsibilities, preferred media, and weekly social media use. Five questions related to attitudes toward social media, specifically focusing on the benefits of information in social media, were taken from a previous study (Li et al. 2018). Participants answered these questions using a 5‐point Likert scale, ranging from 0 (strongly disagree) to 4 (strongly agree). An index score was then calculated using Exploratory Factor Analysis (EFA). The findings from the EFA indicated that these five questions accounted for approximately 71% of the changes in the index.

The initial iteration of the food literacy instrument has undergone a comprehensive evaluation for both validity and reliability within the Portuguese adult demographic (Rosas et al. 2022). This iteration is comprised of five distinct dimensions: culinary skills, production and quality assessment, selection and planning strategies, environmental safety, and origin identification. The reliability of all measurement scales has been substantiated, with Cronbach's alpha coefficients ranging from 0.695 to 0.892. Responses to each individual item are assessed on a scale from 0 to 3, indicating either frequency or levels of agreement. In the context of this study, the Persian adaptation was employed subsequent to its validation (from an earlier investigation) within the Iranian adult population. The participants appraised each item utilizing a 4‐point Likert scale, which corresponded to either the frequency of occurrence (0—never, 1—sometimes, 2—frequently, 3—always) or the degree of agreement (0—completely disagree to 3—completely agree).

### 2.4 Data Analysis

The data were analyzed using SPSS version 26. Quantitative variables were reported as Mean (SD), and qualitative variables were presented as number (%). The comparison of means between groups was performed using an independent *t*‐test. Pearson's correlation coefficients were calculated to detect correlations between parameters. Multiple linear regression analysis with the Stepwise method was conducted to identify predictors of FL. A *p* value below 0.05 was considered significant for testing all hypotheses.

## 3 Results

According to the study results, the average age for women was 33.8 years (SD 10.66) and for men, it was 33.14 years (SD 10.67). Among the total participants, 51.8% were women, 42% were single, and 56.1% were married, while the remaining participants were either divorced or widowed. Of the married individuals, 17.4% did not have children, while the rest had between one and nine children. Additional information can be found in Table 1.

**TABLE 1:** Sociodemographic characteristics of the participants.

Characteristics	Number (%)
Marital status	
Single	230 (42%)
Married	371 (58%)
Educational level	
Lower/upper secondary	58 (10.7%)
Diploma	99 (18.2%)
Postgraduate	52 (9.6%)
Bachelor	226 (41.6%)
Master	86 (15.8%)
PhD	22 (4.1%)
Job	
Employed	154 (29.3%)
Unemployed	16 (3%)
Student	124 (23.6%)
Retired	8 (1.5%)
Worker	95 (18.1%)
Self‐employed	56 (10.6%)
Housewife	59 (11.2%)
Income (Million Rials)	
Under 20	91 (18.4%)
20–40	46 (9.3%)
40–60	41 (8.3%)
Above 60	317 (64%)
Sex	
Female	283 (51.8%)
Male	263 (48.2%)
BMI	
Underweight	29 (5.6%)
Normal	259 (49.7%)
Over weight	166 (31.9%)
Obese	67 (12.9%)
Food‐related responsibility	
Decision/selection	205 (37.6%)
Acquisition/consumption	144 (26.4%)
Preparation	112 (20.6%)
Without role	84 (15.4%)
Preferred media	
Instagram	269 (50.6%)
National	84 (15.8%)
Telegram	39 (7.3%)
Others	140 (26.3%)
Using media (per week)	
1–2	174 (31.9%)
2–3	71 (13%)
3–5	42 (7.7%)
Every day	62 (11.4%)
Never	197 (36.1%)

Table 2 illustrates the participants' food literacy status (total and subscales scores), based on gender. Based on these results, women got higher scores than men in all dimensions (Table 2).

**TABLE 2:** Food literacy status (total and subscales scores), based on gender.

Food literacy	Men	Women	*T*	*p*
	Mean (SD)	Mean (SD)		
Total score	72.30 (9.45)	75.13 (9.86)	3.41	0.001
Skills	27.10 (3.55)	28.10 (3.68)	3.25	0.001
Keeping	20.14 (3.41)	20.77 (3.33)	2.18	0.001
Selection	11.36 (2.16)	11.89 (2.95)	2.33	0.02
Planning	13.68 (2.80)	14.35 (2.86)	2.75	0.006

According to the study results, a relationship was found between food literacy and age (*r* = 0.203, *p* value: 0.000), being female (*t* = 41.3, *p* value: 0.001), and being married (*t* = 4.14, *p* value: 0.001). There was also a positive and significant relationship between FL and the number of children (*r* = 0.165, *p* value: 0.000) and the type of responsibility in making meals (*f* = 9.79, *p* value < 0.001).

The results of the stepwise multiple linear regression analysis indicated that several factors are associated with higher levels of food literacy. These factors include being female (*β* = 0.115, *p* = 0.005), having a higher level of education (*β* = 0.095, *p* = 0.020), being married (*β* = 0.102, *p* = 0.044), greater use of social media (*β* = 0.079, *p* = 0.050), being older (*β* = 0.101, *p* = 0.048), and having a positive attitude toward social media (*β* = 0.294, *p* < 0.001). Together, these predictors accounted for 16.2% of the variance in food literacy (see Table 3).

**TABLE 3:** Linear regression analysis on the relationship between demographic characteristics and food literacy.

Predictors	Unstandardized coefficients		Standardized coefficients	*t*	*p*‐Value
	*B*	Std. error			
(Constant)	51.05	2.475	Beta	20.625	< 0.001
Being female	2.253	0.794	0.115	2.837	0.005
Education	0.685	0.293	0.095	2.335	0.020
Being married	2.024	1.003	0.102	2.017	0.044
Using media	0.450	0.231	0.079	1.951	0.050
Age	0.093	0.047	0.101	1.978	0.048
Attitude	0.532	0.073	0.294	7.260	< 0.001
Model results	*R* = 0.402, Adjusted *R* square = 0.162, *F* = 16.75, *p* < 0.001				

## 4 Discussion

The study looked into the sociodemographic factors that influence food literacy in Iran. It found that age, being female, marital status, number of children, responsibility for food preparation, and use of social media for food‐related information were significantly linked to higher food literacy. On the other hand, there were no significant associations between food literacy and BMI, place of birth, education, income, or occupation. A multivariable regression model showed that positive attitudes toward social media, older age, being female and married, and elevated educational attainment were all linked to higher food literacy. These results highlight that food literacy is influenced by a variety of demographic factors.

In general, the FLW that outlines various domains essential for achieving comprehensive food literacy (Rosas et al. 2021) is affected by sociodemographic factors. This relationship might be explained by the exciting interplay between different aspects of FLW, with some individual and/or social determinants (Rosas et al. 2020). The positive association between age and food literacy aligns with previous research indicating that older individuals often possess extensive knowledge and experience regarding nutrition practices (Koch et al. 2021; Weerasekara et al. 2020). This trend may be attributed to a lifetime of exposure to food‐related decision‐making and health considerations. Older adults typically have more extensive experiences with food preparation and nutrition education, often gained through familial traditions or cultural practices (Michou et al. 2020). As people get older, they often become more concerned about their health, leading them to seek information about healthy eating habits and dietary choices. This increased awareness can help older adults better understand and meet their dietary needs (Bloom et al. 2017; Jacobs et al. 2017).

Furthermore, the finding that women exhibited higher food literacy is consistent with previous studies. It has been shown that women tend to have higher food literacy, which could be due to their traditional roles in household food preparation. In many cultures, including Iran, women are often responsible for meal planning and cooking. This involvement in food‐related activities likely enhances their knowledge of nutrition, cooking, and food safety practices. Additionally, women may have greater access to resources such as cookbooks, nutrition courses, and social networks focused on food‐related topics. As a result, their engagement with food literacy is likely to be more pronounced than that of men (García‐Henche and Cuesta‐Valiño 2022; Savvaidis et al. 2022). Similarly, a study in Saudi Arabia has reported that 46% of parents have poor food literacy. This issue was found to be 2.5 times more prevalent among fathers than mothers (Bookari 2023). The relationship between age and food literacy may be influenced by gender. According to Murakami et al. women tend to enhance their food and cooking skills as they age, whereas the opposite trend was observed for different age groups (Murakami et al. 2022). Marital status can also impact food literacy. Married individuals may be more motivated to learn about nutrition to ensure the health of their families. Research suggests that being married can drive individuals to seek out nutritional information (Zhang et al. 2022).

Research suggests that married individuals tend to become more involved in making dietary decisions for their families. This shift in focus can lead to a greater interest in learning about various foods, meal planning, and using healthy cooking techniques (Tuncil et al. 2018; Voola et al. 2018). The study found a positive correlation between the number of children and food literacy. Parents with children may feel a stronger sense of responsibility to prepare nutritious meals that meet the diverse needs of their family members (Moscato and Machin 2018).

Contrary to the findings in Bookari's study, parents living in crowded families (more children) were reported to have twice the probability of poor food literacy compared to others (Bookari 2023). The variation in alignment or disagreement with other studies might be attributed to the cultural and social disparities between the regions under study, including the attitudes toward food and the significance given to the various aspects of food literacy as a crucial component of a healthy lifestyle. Additionally, it could be related to the role of proper nutrition in preventing or managing diseases.

Research has shown that parents are often motivated to enhance their understanding of nutrition when they have children (Vos et al. 2022). This motivation can arise from a desire to encourage healthy eating habits in children or to prevent diet‐related health issues such as obesity or diabetes (Mahmood et al. 2022). As a result, parents may actively pursue information on nutrition through different channels, such as books, workshops, or online resources, to improve their cooking skills and knowledge (Mahmood et al. 2022; Vos et al. 2022). Taking on the responsibility of meal preparation is associated with higher levels of food literacy. People who regularly prepare meals are more likely to actively engage with nutritional information, which can help them develop a deeper understanding of healthy eating practices and cooking techniques (Amin et al. 2018). For example, those in charge of meal preparation may experiment with different recipes and ingredients in search of healthier alternatives. This hands‐on experience not only improves their cooking skills but also enhances their understanding of nutrition labels and portion sizes (Al Tell et al. 2023; Reicks et al. 2018).

The influence of social media on people's attitudes toward food is clear. A recent study showed that individuals who regularly use social media for food‐related content have higher levels of food literacy. In the past few years, social media platforms have become more influential as sources of nutrition and culinary information (Lewis and Phillipov 2018). Social media platforms offer users a wide range of resources, including cooking tutorials and healthy eating tips tailored to their specific needs. The interactive nature of these platforms encourages users to engage with content that interests them, particularly regarding nutritional information. This reflects the increasing importance of digital platforms as sources of cooking and nutritional advice. Social media can be an effective tool for sharing knowledge about healthy eating, especially among younger individuals who are more likely to regularly use these platforms (Lupton 2018).

Moreover, social media can help in delivering education, especially for those looking to enhance their knowledge about food. Online forums or groups focused on healthy eating can offer support and encouragement, and also encourage discussions about nutrition (Lim et al. 2022). These interactions can boost people's motivation to improve their food and cooking skills (Lewis and Phillipov 2018; Lim et al. 2022; Lupton 2018).

Another study was conducted to gain experts' insight into the factors playing a role in the food business in social media and teenagers' eating behaviors. The study evaluated the teenagers' views on key priorities, challenges, and strategies needed for future policies of this business. This study's results show the existence of a relationship between the content posted in the media and the eating behaviors of teenagers. Teenagers active in this field (social media users) are cognitively vulnerable to the implied tactics of this market. Thus, specific strategies are needed to provide evidence‐based information on social media (van der Bend et al. 2022).

Despite the positive relationships between sociodemographic factors and food literacy, it is important to note that there were no significant associations found for variables such as BMI, birthplace, income, or occupation. This raises important questions about the complexities surrounding food literacy in different contexts. Our research suggests that education can enhance food literacy among populations in Iran. In other words, income may not adequately address practical aspects of nutrition or cooking skills (Krabbe and Lucente 2020).

It is also possible that these findings reflect disparities in access to nutrition education across different regions within the study population. Therefore, it is crucial for policymakers to consider integrating comprehensive nutrition education into the general population, emphasizing practical skills alongside usual food knowledge. The study found that income did not have a significant association with food literacy. While having enough money is important for accessing healthy foods and educational materials about nutrition (Hemmer et al. 2021), our results suggest that even people with lower incomes can have good food literacy. This could be because they are motivated by personal circumstances, such as being responsible for parenting or having health concerns, to make better food choices.

The results of the multiple linear regression in this research indicate that social media use, a positive attitude toward it, higher age and education, being female, and being married are directly associated with the FL score.

The findings have significant implications for public health initiatives in Iran. They suggest that targeted interventions should be developed to improve food literacy among specific demographic groups, such as younger adults and men, who may not be as engaged in food preparation as women. Programs aimed at increasing awareness about the benefits of nutrition literacy could lead to healthier dietary choices and improved public health outcomes. Additionally, using social media for educational campaigns could effectively reach a wider audience. Creating engaging content that promotes healthy eating habits and provides practical cooking tips can help health authorities foster a culture of informed decision‐making regarding food and nutrition.

This study has several strengths, such as its focus on a diverse sample from various regions in Iran and its comprehensive assessment of multiple sociodemographic factors. However, it is important to acknowledge its limitations. The cross‐sectional design limits the ability to draw causal inferences; thus, longitudinal studies are recommended to explore how these associations change over time. Additionally, self‐reported measures may introduce bias; future research should consider using objective assessments of food literacy.

## 5 Conclusion

In conclusion, this study emphasizes the significant sociodemographic determinants of food literacy in Iran. It highlights the roles of age, gender, marital status, number of children, responsibility for meal preparation, and social media use. Understanding these factors is crucial for developing targeted interventions aimed at improving public health outcomes through enhanced food literacy. By promoting informed eating practices across diverse demographic groups, we can contribute to healthier communities in Iran. Future studies should investigate the underlying mechanisms driving the observed associations. Qualitative research could provide deeper insights into how social media influences individual attitudes toward food literacy. Additionally, examining interventions designed to improve food literacy among specific demographics, such as young adults or low‐income families, could yield valuable information on effective strategies for enhancing nutritional knowledge.

## Author Contributions

**Mozhgan Moshtagh:** conceptualization (lead), formal analysis (equal), investigation (equal), methodology (equal), project administration (equal), supervision (lead), writing – original draft (lead), writing – review and editing (lead). **Mojtaba Daneshvar:** formal analysis (equal), investigation (equal), methodology (equal), project administration (equal), software (equal), writing – original draft (equal), writing – review and editing (equal). **Gholamreza Ghaedamini Harouni:** formal analysis (equal), investigation (equal), methodology (equal), project administration (equal), software (equal), writing – review and editing (equal).

## Conflicts of Interest

The authors declare no conflicts of interest.

## Data Availability

The data that support the findings of this study are available upon request from the corresponding author. The data are not publicly available due to privacy restrictions.

## References

[fsn370044-bib-0001] Al Tell, M. , N. Natour , E. Alshawish , and M. Badrasawi . 2023. “The Relationship Between Nutrition Literacy and Nutrition Information Seeking Attitudes and Healthy Eating Patterns Among a Group of Palestinians.” BMC Public Health 23, no. 1: 165. 10.1186/s12889-023-15121-z. 36694185 PMC9875392

[fsn370044-bib-0002] Amin, S. A. , C. Panzarella , M. Lehnerd , S. B. Cash , C. D. Economos , and J. M. Sacheck . 2018. “Identifying Food Literacy Educational Opportunities for Youth.” Health Education & Behavior 45, no. 6: 918–925. 10.1177/1090198118775485. 29848139

[fsn370044-bib-0003] Azevedo Perry, E. , H. Thomas , H. R. Samra , et al. 2017. “Identifying Attributes of Food Literacy: A Scoping Review.” Public Health Nutrition 20, no. 13: 2406–2415. 10.1017/S1368980017001276. 28653598 PMC10261432

[fsn370044-bib-0004] Banna, M. H. A. , M. Hamiduzzaman , S. Kundu , et al. 2022. “The Association Between Bangladeshi Adults' Demographics, Personal Beliefs, and Nutrition Literacy: Evidence From a Cross‐Sectional Survey.” Frontiers in Nutrition 9: 867926. 10.3389/fnut.2022.867926. 35464028 PMC9020226

[fsn370044-bib-0005] Bloom, I. , W. Lawrence , M. Barker , et al. 2017. “What Influences Diet Quality in Older People? A Qualitative Study Among Community‐Dwelling Older Adults From the Hertfordshire Cohort Study, UK.” Public Health Nutrition 20, no. 15: 2685–2693. 10.1017/S1368980017001203. 28724471 PMC5612401

[fsn370044-bib-0006] Bolek, S. 2020. “Consumer Knowledge, Attitudes, and Judgments About Food Safety: A Consumer Analysis.” Trends in Food Science and Technology 102: 242–248. 10.1016/j.tifs.2020.03.009.

[fsn370044-bib-0007] Bookari, K. 2023. “A Cross‐Sectional Exploratory Study of Food Literacy Among Saudi Parents of Adolescent Children Aged 10 to 19 Years.” Frontiers in Nutrition 9: 1083118. 10.3389/fnut.2022.1083118. 36687688 PMC9853415

[fsn370044-bib-0008] Fathelrahman, E. , S. Muhammad , and U. Al‐Ain . 2016. “Food Policy and Food Security Challenges in the Middle East and North Africa Region.” In Reference Module in Food Science, 3304–3308. Elsevier. 10.1016/B978-0-08-100596-5.03308-4.

[fsn370044-bib-0009] Forray, A. I. , M. A. Coman , R. M. Cherecheș , and C. M. Borzan . 2023. “Exploring the Impact of Sociodemographic Characteristics and Health Literacy on Adherence to Dietary Recommendations and Food Literacy.” Nutrients 15, no. 13: 853. 10.3390/nu15132853. 37447180 PMC10343671

[fsn370044-bib-0010] García‐Henche, B. , and P. Cuesta‐Valiño . 2022. “The Increasing Visibility of Women in Gastronomy.” International Journal of Gastronomy and Food Science 30: 100589. 10.1016/j.ijgfs.2022.100589.

[fsn370044-bib-0011] Gibbs, H. , and K. Chapman‐Novakofski . 2012. “A Review of Health Literacy and Its Relationship to Nutrition Education.” Topics in Clinical Nutrition 27, no. 4: 325–333.

[fsn370044-bib-0012] Hemmer, A. , K. Hitchcock , Y. S. Lim , M. Butsch Kovacic , and S.‐Y. Lee . 2021. “Development of Food Literacy Assessment Tool Targeting Adults With Low Income.” Journal of Nutrition Education and Behavior 53, no. 11: 966–976. 10.1016/j.jneb.2021.05.007. 34426065 PMC8922242

[fsn370044-bib-0013] Jacobs, W. , A. O. Amuta , and K. C. Jeon . 2017. “Health Information Seeking in the Digital Age: An Analysis of Health Information Seeking Behavior Among US Adults.” Cogent Social Science 3, no. 1: 1302785. 10.1080/23311886.2017.1302785.

[fsn370044-bib-0014] Koch, F. , I. Hoffmann , and E. Claupein . 2021. “Types of Nutrition Knowledge, Their Socio‐Demographic Determinants and Their Association With Food Consumption: Results of the NEMONIT Study.” Frontiers in Nutrition 8: 14. 10.3389/fnut.2021.630014. PMC7907003 33644108

[fsn370044-bib-0015] Krabbe, J. , and M. Lucente . 2020. “Demographic Variation in Nutrition Knowledge Among Chiropractic Students.” Nutritional Perspectives: Journal of the Council on Nutrition 43, no. 1: 33–40.

[fsn370044-bib-0016] Lewis, T. , and M. Phillipov . 2018. “Food/Media: Eating, Cooking, and Provisioning in a Digital World.” Communication Research and Practice 4, no. 3: 207–211. 10.1080/22041451.2018.1482075.

[fsn370044-bib-0017] Li, Y. , X. Wang , X. Lin , and M. Hajli . 2018. “Seeking and Sharing Health Information on Social Media: A Net Valence Model and Cross‐Cultural Comparison.” Technological Forecasting and Social Change 126: 28–40. 10.1016/j.techfore.2016.07.021.

[fsn370044-bib-0018] Lim, M. S. C. , A. Molenaar , L. Brennan , M. Reid , and T. McCaffrey . 2022. “Young Adults' Use of Different Social Media Platforms for Health Information: Insights From Web‐Based Conversations.” Journal of Medical Internet Research 24, no. 1: e23656. 10.2196/23656. 35040796 PMC8808344

[fsn370044-bib-0019] Lupton, D. 2018. “Cooking, Eating, Uploading: Digital Food Cultures.” In Bloomsbury Handbook of Food and Popular Culture, 66–79. Bloomsbury.

[fsn370044-bib-0020] Mahmood, L. , L. A. Moreno , P. Flores‐Barrantes , et al. 2022. “Parental Food Consumption and Diet Quality and Its Association With Children's Food Consumption in Families at High Risk of Type 2 Diabetes: The Feel4Diabetes‐Study.” Public Health Nutrition 25, no. 12: 3344–3355. 10.1017/S1368980022002245. PMC9991723 36217747

[fsn370044-bib-0021] McKay, F. H. , B. C. Haines , and M. Dunn . 2019. “Measuring and Understanding Food Insecurity in Australia: A Systematic Review.” International Journal of Environmental Research and Public Health 16, no. 3: 476. 10.3390/ijerph16030476. 30736305 PMC6388276

[fsn370044-bib-0022] Michou, M. , D. B. Panagiotakos , C. Lionis , and V. Costarelli . 2020. “Sex and Age in Relation to Health and Nutrition Literacy Levels in a Sample of Greek Adults.” International Journal of Health Promotion and Education 58, no. 5: 229–241. 10.1080/14635240.2019.1681289.

[fsn370044-bib-0023] Moscato, E. M. , and J. E. Machin . 2018. “Mother Natural: Motivations and Associations for Consuming Natural Foods.” Appetite 121: 18–28. 10.1016/j.appet.2017.10.031. 29080704

[fsn370044-bib-0024] Mozaffarian, D. 2016. “Dietary and Policy Priorities for Cardiovascular Disease, Diabetes, and Obesity.” Circulation 133, no. 2: 187–225. 10.1161/CIRCULATIONAHA.115.018585. 26746178 PMC4814348

[fsn370044-bib-0025] Murakami, K. , N. Shinozaki , X. Yuan , et al. 2022. “Food Choice Values and Food Literacy in a Nationwide Sample of Japanese Adults: Associations With Sex, Age, and Body Mass Index.” Nutrients 14, no. 9: 1899. 10.3390/nu14091899. 35565865 PMC9102665

[fsn370044-bib-0026] Reicks, M. , M. Kocher , and J. Reeder . 2018. “Impact of Cooking and Home Food Preparation Interventions Among Adults: A Systematic Review (2011–2016).” Journal of Nutrition Education and Behavior 50, no. 2: 148–172. 10.1016/j.jneb.2017.08.004. 28958671

[fsn370044-bib-0027] Rosas, R. , F. Pimenta , I. Leal , and R. Schwarzer . 2020. “FOODLIT‐PRO: Food Literacy Domains, Influential Factors and Determinants—A Qualitative Study.” Nutrients 12, no. 1: 88. 10.3390/nu12010088. PMC7019603 31892245

[fsn370044-bib-0028] Rosas, R. , F. Pimenta , I. Leal , and R. Schwarzer . 2021. “FOODLIT‐PRO: Conceptual and Empirical Development of the Food Literacy Wheel.” International Journal of Food Sciences and Nutrition 72, no. 1: 99–111. 10.1080/09637486.2020.1762547. 32397776

[fsn370044-bib-0029] Rosas, R. , F. Pimenta , I. Leal , and R. Schwarzer . 2022. “FOODLIT‐Tool: Development and Validation of the Adaptable Food Literacy Tool Towards Global Sustainability Within Food Systems.” Appetite 168: 105658. 10.1016/j.appet.2021.105658. 34461194

[fsn370044-bib-0030] Savvaidis, I. N. , A. Al Katheeri , S.‐H. E. Lim , K.‐S. Lai , and A. Abushelaibi . 2022. “Chapter 1—Traditional Foods, Food Safety Practices, and Food Culture in the Middle East.” In Food Safety in the Middle East, edited by I. N. Savvaidis and T. M. Osaili , 1–31. Academic Press.

[fsn370044-bib-0031] Soon, J. M. , I. Vanany , I. R. Abdul Wahab , R. H. Hamdan , and M. H. Jamaludin . 2021. “Food Safety and Evaluation of Intention to Practice Safe Eating Out Measures During COVID‐19: Cross Sectional Study in Indonesia and Malaysia.” Food Control 125: 107920. 10.1016/j.foodcont.2021.107920. 35668872 PMC9159731

[fsn370044-bib-0032] Thomas, H. , E. Azevedo Perry , J. Slack , et al. 2019. “Complexities in Conceptualizing and Measuring Food Literacy.” Journal of the Academy of Nutrition and Dietetics 119, no. 4: 563–573. 10.1016/j.jand.2018.10.015. 30670348

[fsn370044-bib-0033] Tuncil, E. , G. Arman , and M. Fisunoglu . 2018. “The Role of Gender and Marital Status on Food Choice.” Clinical Nutrition 37: S130. 10.1016/j.clnu.2018.06.1488.

[fsn370044-bib-0034] van der Bend, D. L. M. , T. Jakstas , E. van Kleef , V. A. Shrewsbury , and T. Bucher . 2022. “Making Sense of Adolescent‐Targeted Social Media Food Marketing: A Qualitative Study of Expert Views on Key Definitions, Priorities and Challenges.” Appetite 168: 105691. 10.1016/j.appet.2021.105691. 34509544

[fsn370044-bib-0035] Vidgen, H. A. , and D. Gallegos . 2014. “Defining Food Literacy and Its Components.” Appetite 76: 50–59. 10.1016/j.appet.2014.01.010. 24462490

[fsn370044-bib-0036] Voola, A. P. , R. Voola , J. Wyllie , J. Carlson , and S. Sridharan . 2018. “Families and Food: Exploring Food Well‐Being in Poverty.” European Journal of Marketing 52, no. 12: 2423–2448. 10.1108/EJM-10-2017-0763.

[fsn370044-bib-0037] Vos, M. , B. Deforche , A. Van Kerckhove , et al. 2022. “Determinants of Healthy and Sustainable Food Choices in Parents With a Higher and Lower Socioeconomic Status: A Qualitative Study.” Appetite 178: 106180. 10.1016/j.appet.2022.106180. 35863506

[fsn370044-bib-0038] Weerasekara, P. C. , C. R. Withanachchi , G. A. S. Ginigaddara , and A. Ploeger . 2020. “Food and Nutrition‐Related Knowledge, Attitudes, and Practices Among Reproductive‐Age Women in Marginalized Areas in Sri Lanka.” International Journal of Environmental Research and Public Health 17, no. 11: 3985. 10.3390/ijerph17113985. 32512750 PMC7312908

[fsn370044-bib-0039] Whittall, B. , S. M. Warwick , D. J. Guy , and K. M. Appleton . 2023. “Public Understanding of Sustainable Diets and Changes Towards Sustainability: A Qualitative Study in a UK Population Sample.” Appetite 181: 106388. 10.1016/j.appet.2022.106388. 36414148

[fsn370044-bib-0040] Wijayaratne, S. P. , M. Reid , K. Westberg , A. Worsley , and F. Mavondo . 2018. “Food Literacy, Healthy Eating Barriers and Household Diet.” European Journal of Marketing 52, no. 12: 2449–2477. 10.1108/EJM-10-2017-0760.

[fsn370044-bib-0041] Zhang, Y. , Z. Zhang , M. Xu , et al. 2022. “Development and Validation of a Food and Nutrition Literacy Questionnaire for Chinese Adults.” Nutrients 14, no. 9: 1933. 10.3390/nu14091933. 35565900 PMC9104569

